# Inhibitory luminopsins: genetically-encoded bioluminescent opsins for versatile, scalable, and hardware-independent optogenetic inhibition

**DOI:** 10.1038/srep14366

**Published:** 2015-09-24

**Authors:** Jack K. Tung, Claire-Anne Gutekunst, Robert E. Gross

**Affiliations:** 1 Coulter Department of Biomedical Engineering, Georgia Institute of Technology and Emory University, Atlanta, GA; 2 Department of Neurosurgery, Emory University, Atlanta, GA; 3 Department of Neurology, Emory University, Atlanta, GA.

## Abstract

Optogenetic techniques provide an unprecedented ability to precisely manipulate neural activity in the context of complex neural circuitry. Although the toolbox of optogenetic probes continues to expand at a rapid pace with more efficient and responsive reagents, hardware-based light delivery is still a major hurdle that limits its practical use *in vivo*. We have bypassed the challenges of external light delivery by directly coupling a bioluminescent light source (a genetically encoded luciferase) to an inhibitory opsin, which we term an inhibitory luminopsin (iLMO). iLMO was shown to suppress action potential firing and synchronous bursting activity *in vitro* in response to both external light and luciferase substrate. iLMO was further shown to suppress single-unit firing rate and local field potentials in the hippocampus of anesthetized rats. Finally, expression of iLMO was scaled up to multiple structures of the basal ganglia to modulate rotational behavior of freely moving animals in a hardware-independent fashion. This novel class of optogenetic probes demonstrates how non-invasive inhibition of neural activity can be achieved, which adds to the versatility, scalability, and practicality of optogenetic applications in freely behaving animals.

Optogenetic techniques have revolutionized the field of neuroscience because they have given scientists the ability to selectively activate or inhibit neural activity in the context of exquisitely complex neural circuitry^1^. These techniques rely on the use of light-sensitive ion channels or pumps (opsins), which are expressed in a cell-type specific manner in the brain and activated by an external light source such as a laser or light-emitting diode (LED).

Although optogenetic techniques generally work well in small rodents, several technical challenges with light delivery into the brain still need to be addressed before these techniques can be routinely utilized in freely behaving animals or translated into larger and more complex animal models. The most common solution for delivering light into the brain is via a surgically implanted optical fiber coupled to an external light source^2^. Not only do these chronically implanted optical fibers pose risks for infection and tissue damage, but they also raise practical impediments (*e.g*. limited range of movement or need for extra hardware such as optical commutators) for conducting experiments with freely moving animals. Transmission of external light through the brain is also extremely inefficient due to light scatter and tissue absorption^3^,^4^. In fact, most of the light emitted from an implanted fiber optic is completely attenuated within 1 mm of the fiber tip^5^. Optogenetic applications in other tissues (*e.g*. heart^6^ and peripheral nerves^7^) also share similar challenges with light delivery, where light scatter and attenuation can be a significant problem^8^. While progress has been made towards developing optogenetic tools that require less light (*i.e.* more sensitive and red-shifted opsins^9^,^10^,^11^), the scalability of this approach to larger structures of non-human primates or human patients is still unclear.

We have bypassed the challenges of external light delivery by directly coupling bioluminescent proteins to conventional light-sensitive opsins. Specifically, we have utilized luciferase enzymes (which emit bioluminescence in the presence of a chemical substrate, luciferin) as an alternative light source for activating light-sensitive opsins. Since luciferase enzymes can be genetically encoded and expressed together with opsins in a cell-type specific manner, these bioluminescent light sources obviate any need for chronic implants or external hardware for delivery of light into the brain. A genetically encoded light source also offers much versatility in illuminating large, multiple, or complex structures in the brain due to the ability to scale up expression in a cell-type dependent manner.

Building upon the previously demonstrated feasibility of coupling bioluminescent proteins to excitatory channelrhodopsins in a single fusion protein termed a luminosopin (LMO)^12^, we describe here a new class of ***inhibitory luminopsins*** (**iLMOs**) consisting of *Renilla* luciferase (Rluc) and *Natronomonas* halorhodopsin (NpHR) and demonstrate its ability to silence neural activity *in vitro* and *in vivo* in response to both external light and chemical substrate. This new class of optogenetic probes not only allows for optogenetic inhibition without external hardware, but also permits multi-modal (i.e. optical and chemical) methods of neuromodulation that can result in varying temporal effects.

Here we demonstrate how the versatility of conventional light-sensitive opsins can be increased when they are converted into luminopsins, enabling a readily scalable and non-invasive means of optogenetic manipulation that may have unique advantages over other chemical-genetic approaches.

## Results

### Design and construction of inhibitory luminopsins (iLMOs)

**Figure 1:**
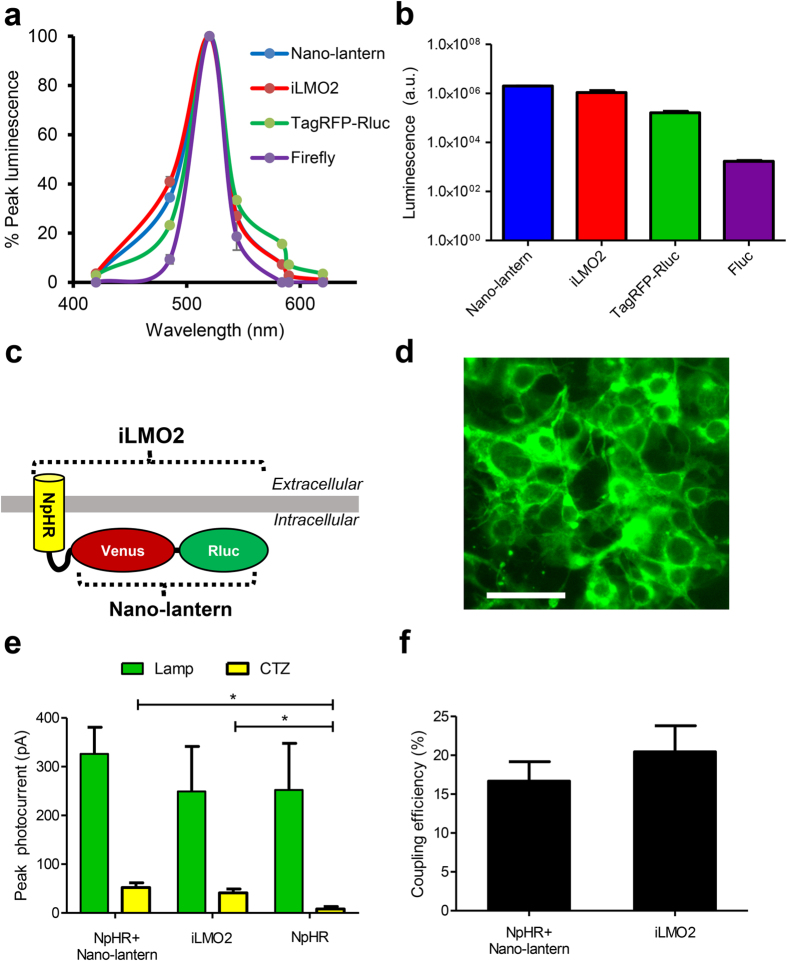
NpHR can be activated by both external light and luciferase-derived bioluminescence in HEK293 cells expressing iLMO2. (**a**) Emission spectra and (**b**) Total luminescence measured from transfected HEK293 cells expressing various luciferases (Nano-lantern, TagRFP-Rluc, Firefly) and iLMO2. (**c**) Schematic representation of the iLMO2 fusion protein. (**d**) Fluorescence image showing membrane-localized expression of iLMO2 in transfected HEK293 cells. Scale bar: 50 μm (**e**) Average peak photocurrent responses to green lamp illumination and CTZ measured from HEK293 cells transfected with NpHR and Nano-lantern separately (NpHR + Nano-lantern), iLMO2 fusion protein, or NpHR alone (n = 5 for each group). Mean responses to CTZ were significantly different (*p < 0.05) but responses to green lamp illumination were not (p > 0.05) by one-way ANOVA with Bonferroni posthoc test. (**f**) Coupling efficiency (peak photocurrent from CTZ divided by peak photocurrent from lamp) of transfected HEK293 cells expressing NpHR and Nano-lantern separately (NpHR + Nano-lantern) (n = 5) and iLMO2 fusion protein (n = 5). Error bars indicate standard error of the mean.

In search of a luciferase most suitable for activating NpHR, we characterized bioluminescence from several luciferase proteins that have compatible emission spectra with NpHR: a red-shifted *Renilla* luciferase (TagRFP-RLuc^13^), a brighter *Renilla* luciferase variant (Nano-lantern^14^), and firefly luciferase (FLuc^15^). HEK293 cells were transiently transfected with these luciferases and were characterized in a plate reader by measuring bioluminescence intensity and emission spectrum. The emission spectra for all the luciferases peaked at ~525-nm, with TagRFP-RLuc and Nano-lantern having a wider emission spectrum compared to Fluc (Fig. 1a) due to bioluminescence resonance energy transfer (BRET)^16^,^17^. Although the red-shifted emission spectrum of TagRFP-RLuc appeared to be more preferable for activating NpHR, total luminescence from TagRFP-RLuc was significantly lower than that of Nano-lantern by a factor of 12.3 (p < 0.001 by one-way ANOVA with Bonferroni posthoc test; *n *= 3 for each group) (Fig. 1b). Fluc did not appear to be a suitable luciferase because it utilizes ATP (which could directly influence neural activity^18^) and generates bioluminescence that is several orders of magnitude lower than the RLuc variants. We therefore decided to couple TagRFP-Rluc and Nano-lantern to NpHR by cloning each luciferase to the 3′ end of NpHR to create two fusion proteins, which we termed iLMO1 and iLMO2 (Fig. 1c), respectively. Endoplasmic reticulum export sequences described by Gradinaru *et al.*^19^ were also included at the 3′ end of the fusion protein to improve membrane trafficking. We present the results for iLMO2 here since it was able to produce more robust effects.

### *In vitro* characterization of iLMO2 in HEK293 cells

We first examined the bioluminescence from iLMO2 and compared it to that of Nano-lantern alone in transfected HEK293 cells to see whether the creation of the fusion protein had altered the functionality of the luciferase. Both the bioluminescence intensity and emission spectrum of iLMO2 were not significantly different from that of Nano-lantern alone (Fig. 1a,b), which suggests that the functionality of the luciferase moiety is preserved in the iLMO2 fusion protein.

Next, we evaluated the functionality of the NpHR moiety in iLMO2. Based on the localization of the fluorescence tag, HEK293 cells transfected with iLMO2 efficiently expressed the fusion protein in the cell membrane (Fig. 1d). Whole-cell patch clamp recordings revealed that iLMO2-expressing HEK293 cells produced hyperpolarizing outward currents in response to green lamp illumination (Fig. 1e), which was not significantly different in peak amplitude from cells expressing NpHR alone or from cells co-expressing NpHR with Nano-lantern (*p *> 0.05; one-way ANOVA with Bonferroni posthoc test; *n *= 5 for each group). These results therefore indicated that NpHR functionality was not significantly affected by fusion or co-expression with Nano-lantern.

We next tested the capability of luciferase-driven activation of NpHR in iLMO2-expressing HEK293 cells. Application of coelenterazine (CTZ), the substrate for *Renilla* luciferase, to cells expressing iLMO2 generated outward currents that were significantly greater than the negligible CTZ-induced currents produced by cells expressing NpHR alone (*p *< 0.05; one-way ANOVA with Bonferroni posthoc test; *n *= 5 for each group), indicating that the photocurrents measured in iLMO2 expressing cells were specifically mediated by bioluminescence generated in the presence of CTZ (Fig. 1e). Interestingly, the response of iLMO2-expressing cells to CTZ was not significantly different from cells co-expressing Nano-lantern and NpHR separately, which suggests that bioluminescence from Nano-lantern can activate NpHR whether or not they are physically coupled together.

To assess the efficiency of bioluminescence-driven activation of NpHR, the coupling efficiency (peak CTZ-induced photocurrent divided by peak lamp-induced photocurrent) was calculated. The mean coupling efficiency was higher when Nano-lantern and NpHR were expressed together as the iLMO2 fusion protein compared to when they were co-expressed separately (Fig. 1f), but this difference was not statistically significant (*p *> 0.05; one-tailed Student’s *t*-test, n = 5 for each group).

These results demonstrate that *Renilla* luciferase can activate NpHR when they are either co-expressed or coupled together as a single fusion protein. Although co-expression of opsin and luciferase has been shown to be technically feasible by us and others^20^, the single fusion protein approach generated more robust responses and provided a more practical means of gene delivery.

### iLMO2 is able to suppress neural activity *in vitro*

**Figure 2:**
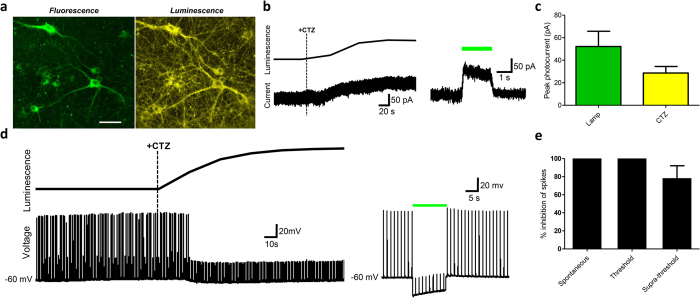
iLMO2 is able to suppress action potential firing *in vitro*. (**a**) Left: fluorescence micrograph depicting dissociated cortical neurons expressing iLMO2. Right: bioluminescence image taken in the same field of view after addition of CTZ. Scale bar: 20 μm. (**b**) Representative voltage clamp recordings of a neuron expressing iLMO2 demonstrate hyperpolarizing outward photocurrents in response to CTZ (left, dashed line indicates time of CTZ addition) and green lamp illumination (right, green bar denotes period of illumination). Note that the outward current induced by CTZ coincides with an increase in luminescence (top left). (**c**) Average peak photocurrent response to CTZ and green lamp illumination in neurons expressing iLMO2 fusion protein (n = 8). (**d**) Representative current clamp recordings from neurons expressing iLMO2 demonstrate complete suppression of action potentials (evoked by 1 Hz threshold-level current injections) in response to CTZ (left, dashed line indicates time of CTZ addition) and green lamp illumination (right, green bar denotes period of illumination). A sustained hyperpolarizing response coincides with an increase in luminescence after CTZ addition. (**e**) Average percent inhibition of spontaneous (n = 3) and evoked (n = 6 for threshold-level current injections; n = 4 for supra-threshold) action potentials in cortical neurons expressing iLMO2. Error bars indicate standard error of the mean.

To express iLMO2 in neurons, the iLMO2 cassette was packaged into a lentiviral vector and used to transduce dissociated cortical neurons *in vitro*. Fluorescence micrographs showed strong membrane-localized expression of iLMO2 (Fig. 2a, left) with no obvious morphological signs of toxicity. After addition of CTZ, robust bioluminescence signals were detected and could be imaged with resolution comparable to that of fluorescence imaging (Fig. 2a, right). Whole-cell patch clamp recordings showed that neurons expressing iLMO2 generated hyperpolarizing outward currents in response to both external lamp illumination and CTZ application (Fig. 2b,c). Although external lamp illumination was able to generate larger photocurrents than CTZ application, the difference was not statistically significant (*p *> 0.05; two-tailed paired Student’s *t*-test, n = 8 for each group). The time course of the CTZ-induced photocurrents corresponded to the bioluminescence signal simultaneously detected from the same cells, suggesting that the outward currents seen after CTZ application were due to luciferase activity. These effects were not directly caused by CTZ itself because negligible photocurrent responses were measured when CTZ was added to non-transduced cells. The coupling efficiency of iLMO2 in cortical neurons was 72.6 ± 14.4% (mean ± SEM; *n *= 8), which was significantly higher than that found in HEK293 cells (*p* < 0.05; one-tailed Student’s *t*-test; *n *= 5).

Next, iLMO2 was tested in the context of suppressing both spontaneous and evoked action potentials in dissociated cortical neuron cultures. Similar to external lamp illumination (Fig. 2d, right), CTZ application was able to completely inhibit action potentials evoked by threshold-level (rheobase levels where action potentials are evoked ~50% of the time) current injections given at 1 Hz (Fig. 2d, left). In addition, iLMO2 was able to completely suppress spontaneous action potential firing after CTZ application (Fig. 2e). In contrast, action potentials evoked by supra-threshold (action potentials evoked 100% of the time) current injections were only partially suppressed by CTZ (Fig. 2e), which was most likely due to the inability of iLMO2 to overcome the relatively larger inward current injections. These experiments corroborate our previous findings in HEK293 cells and demonstrate that iLMO2 can inhibit neuronal firing in single cells in response to both external light and luciferase substrate.

**Figure 3:**
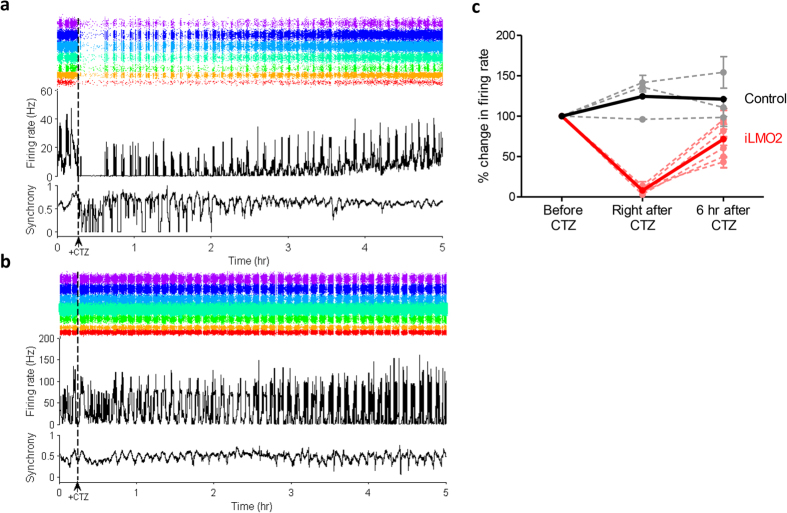
iLMO2 is able to suppress synchronous bursting activity *in vitro*. (**a**) A representative multielectrode array recording of a culture transduced with iLMO2 fusion protein shows complete inhibition of spontaneous bursting activity after addition of CTZ (dashed line). Spontaneous bursting activity eventually returns to baseline levels over a period of several hours. In both (**a**) and (**b**) the middle trace shows array-wide firing rate, top trace shows corresponding raster (each color corresponds to a different electrode), and bottom trace depicts synchrony across two random electrodes. (**b**) Representative multielectrode array recording of a sister control culture (un-transduced) showing no effect of CTZ on spontaneous bursting activity. (**c**) Average change in multi-unit firing rate (9 channels per culture) of 10 min intervals before, immediately after, and 6 hours after addition of CTZ in iLMO2 expressing (dotted red lines, n = 6) and sister control cultures (dotted black lines, n = 3). Solid bold lines represent averages for each group of cultures. Error bars indicate standard error of the mean.

To test the ability of iLMO2 to silence neuronal activity of a population of neurons, we cultured cortical neurons on multielectrode arrays (MEAs) and transduced them with iLMO2 lentivirus. When cortical neurons are cultured on MEAs at high density, they spontaneously exhibit synchronous burst-firing activity across the culture^21^,^22^. Addition of CTZ inhibited burst-firing activity in a period of minutes, resulting in near-zero total firing rate across all the electrodes in the array (Fig. 3a). This effect persisted for up to 20 minutes before bursts returned and total firing rate gradually recovered back to baseline levels. Recovery back to baseline required several hours, which is consistent with the amount of time these cultures take to recover from transient suppression of bursting activity^23^. The synchrony (a measure of concurrent spike times across electrodes) of cultures expressing iLMO2 also transiently decreased during periods of suppressed burst-firing, confirming that CTZ was suppressing synchronous bursting activity. These changes induced by CTZ were not seen in sister control cultures that were not transduced with iLMO2 lentivirus (Fig. 3b), indicating that CTZ was specifically acting through iLMO2. On average, multiunit activity in each electrode of the array slightly increased in control cultures after addition of CTZ (Fig. 3c, black trace), whereas cultures expressing iLMO2 showed an unequivocal marked reduction in multiunit activity in each electrode of the array after addition of CTZ (Fig. 3c, red trace). These results suggest that iLMO2 is able to suppress synchronous bursting activity across the entire neural network.

### iLMO2 is able to suppress neural activity *in vivo*

**Figure 4:**
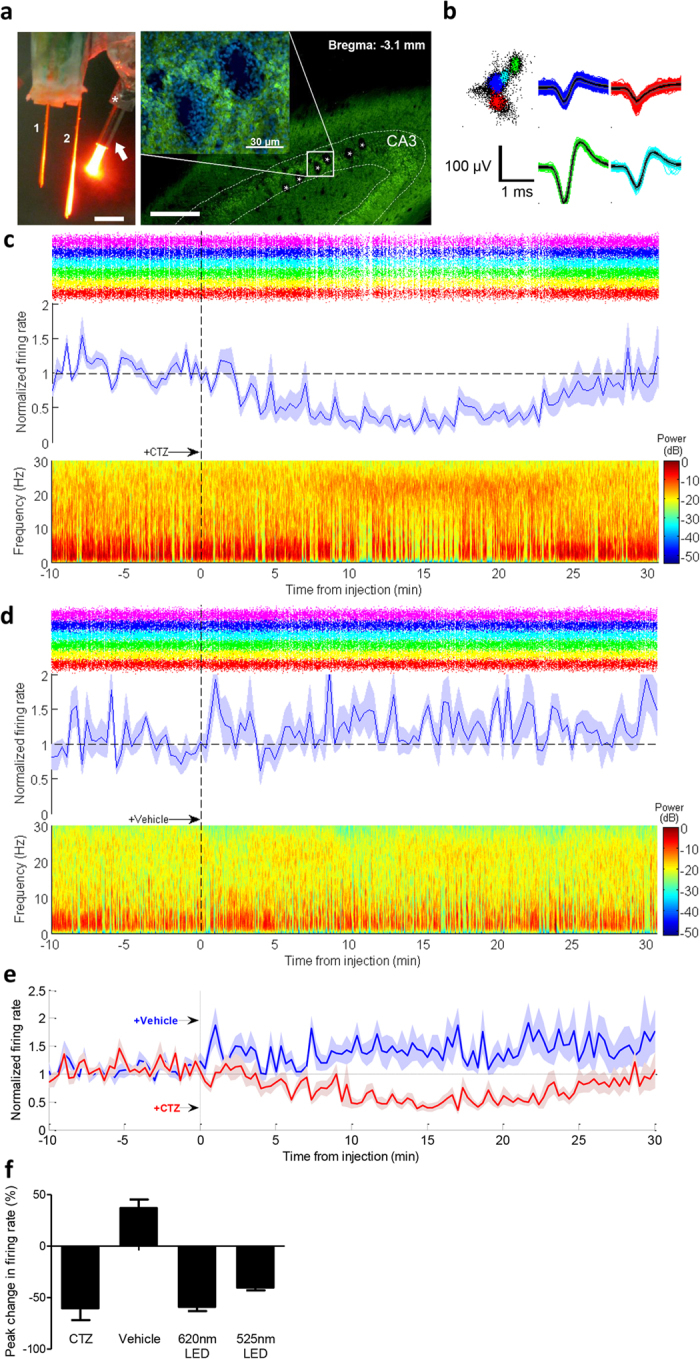
iLMO2 is able to suppress neural activity in anesthetized rats. (**a**) Left: photograph depicting a cannula-electrode in which a guide cannula (white asterisk) was glued to a 16-channel microwire array (row 1 targeting CA1, row 2 targeting CA3) so that an optical fiber inserted through an injection cannula (white arrow) can be positioned 1–2 mm away from electrode tips. Scale bar: 1 mm. Right: Histology depicting tips of electrode tracks (asterisks) targeted to the pyramidal cell layer (outlined) of the dorsal hippocampus. Note robust expression of iLMO2 (green) in the pyramidal cells around electrode holes. DAPI stain (blue) highlights nuclei of migratory glial cells around electrode tips. Scale bar: 100 μm. (**b**) Example of single-units sorted using super-paramagnetic clustering. (**c**) Population average (n = 6) of normalized single-unit firing rate over time (middle, SEM shaded) with corresponding raster (top, each color corresponds to different unit) and spectrogram (bottom) for a representative CTZ injection trial (injected at time = 0, vertical dashed line). (**d**) Population average (n = 6) of normalized single-unit firing rate, raster, and spectrogram for the same units shown in (**c**) for a representative vehicle injection trial (injected at time = 0, vertical dashed line). (**e**) Normalized single-unit firing rate averaged across all intracerebral CTZ (red) and vehicle (blue) injection trials (n = 5 trials each, same 24 units for each group) from the same animal shown above. Injections occurred at t = 0 denoted by vertical dashed line. (**f**) Average peak change in single-unit firing rate to light, CTZ, and vehicle across all animals (n = 3). Error bars indicate standard error of the mean.

Next, we examined the ability of iLMO2 to suppress neural activity in anesthetized or awake rats. The iLMO2 cassette was cloned into an adeno-associated viral (AAV) vector under the control of the CaMKIIα promoter, and the virus was stereotaxically injected into the dorsal hippocampus of rats to selectively target expression of iLMO2 to pyramidal cells of CA3 and CA1 (Fig. 4a, right). A cannula-electrode consisting of a 16-channel microwire array and a guide cannula (Fig. 4a, left) was fabricated and subsequently implanted in the same area, where each row of the array targeted a different pyramidal cell layer of CA3 and CA1. The cannula-electrode was then chronically implanted, which allowed for easy insertion of an injection cannula or optical fiber for intracerebral injections and optical stimulation, respectively, to the same area in each animal over multiple trials under anesthesia. Before each injection trial, single-units whose firing rate was significantly reduced by conventional photostimulation (via 525-nm or 620-nm LED) were first identified for subsequent single-unit analysis. The optical fiber was then replaced with an injection cannula that contained either 2 μl of CTZ or vehicle. When CTZ was injected intracerebrally, single-unit firing rate (of those units that responded previously to photostimulation) decreased and reached peak inhibition about 15 minutes after injection (Fig. 4c, top and middle). The firing rate subsequently returned back towards baseline levels over the next 15 minutes. In addition to reducing single-unit firing rate, CTZ also reduced the low frequency power (1–10 Hz) in the local field potential of the same electrodes (Fig. 4c, bottom), indicating that the dominant theta rhythms of the hippocampus^24^ were being suppressed. In contrast, when vehicle was injected in the same animal as a control, single-unit firing rate increased and no change in the local field potential was observed (Fig. 4d). These trials of intracerebral CTZ and vehicle injections were repeated daily with an alternating order over a period of 5–7 days and averaged for each animal (Fig. 4e). The average peak response to CTZ was comparable to conventional photostimulation with LEDs: single-unit firing rate was reduced on average by 52.1 ± 5.4%, 37.5 ± 6.9%, and 45.0 ± 4.6% (mean ± SEM; *n *= 24 units each) by 620-nm light, 525-nm light, and CTZ, respectively (Supplementary Figure 1). On average, vehicle injections increased single-unit firing rate by 44.6 ± 9.6% (mean ± SEM; *n *= 24 units). Across all animals tested, the average peak inhibition of single-unit activity was 60.3 ± 11.56% by intracerebral CTZ injection, 58.9 ± 4.18% by 620-nm light, and 40.2 ± 2.7% by 525-nm green light (mean ± SEM, *n *= 3 animals; Fig. 4f). CTZ was thus able to suppress neural activity on both a cellular and network level *in vivo* and its efficacy was comparable to conventional photostimulation through a fiber optic.

**Figure 5:**
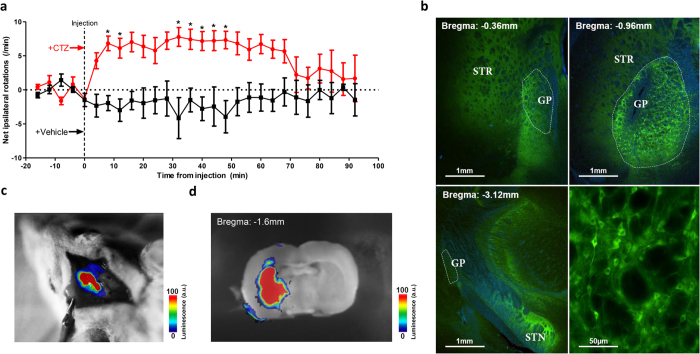
iLMO2 can be expressed in multiple structures of the basal ganglia and is able to induce ipsilateral rotation in awake rats. (**a**) Intravenous administration of CTZ was able to significantly increase net ipsilateral rotations over mean baseline rate in rats unilaterally expressing iLMO2 in the striatum and globus pallidus. CTZ or vehicle was injected through a jugular venous catheter at t = 0 (dashed line). The response to CTZ was significantly different than vehicle by two-way ANOVA with Bonferroni posthoc test (*p < 0.05; n = 5 trials for CTZ, n = 6 trials for vehicle across 3 animals). (**b**) Expression of iLMO2 (green) was found throughout the entire striatum and globus pallidus (STR-striatum, GP-globus pallidus is outlined). Neurons in the globus pallidus expressed iLMO2 (bottom right) and sent labeled projections to the subthalamic nucleus (STN, bottom left). Blue: DAPI. (**c**) Bioluminescence image of a rat expressing iLMO2 after CTZ was administered intravenously under anesthesia. (**d**) Bioluminescence image of an acute brain slice showing activation of iLMO2 throughout the striatum and globus pallidus after addition of CTZ. Color bar indicates relative luminescence intensity.

Lastly, we tested the ability of iLMO2 to modulate rotational behavior in awake behaving rats. Unlike conventional optogenetic stimulation, luminopsins do not require external light delivery and can instead be activated by systemic CTZ administration, facilitating long-term studies of freely behaving animals in a non-invasive manner. Unilateral lesions of the globus pallidus in rats have been demonstrated to produce a consistent and predictable increase in ipsilateral rotation after administration of stimulants such as amphetamine or apomorphine^25^. We therefore sought to mimic these motor effects with optogenetic inhibition of the globus pallidus with iLMO2. AAV encoding iLMO2 was unilaterally injected into the globus pallidus of rats, and two weeks later they underwent multiple amphetamine-induced rotation tests. For each test, a baseline recording of at least 10 minutes was first obtained before the animal was intravenously injected with either CTZ or vehicle through a jugular venous catheter. CTZ injections resulted in a significant increase in mean net ipsilateral rotations above the baseline (Fig. 5a, red trace). The effect of CTZ persisted for more than 1 hour before rotational preference returned to baseline. When vehicle was injected in the same animal, such an effect was not observed (Fig. 5a, black trace). To confirm the activation of iLMO2 upon CTZ administration, *in vivo* bioluminescence imaging was conducted in these animals after conclusion of the rotation experiments. After intravenous injection of CTZ, a robust bioluminescence signal was detected through a craniotomy above the virus injection site (Fig. 5c), confirming that CTZ was crossing the blood-brain-barrier and reaching cells in the brain expressing iLMO2. These results also demonstrate a convenient means to functionally detect and confirm expression of iLMOs in deep structures of the brain. To estimate the extent of activation of iLMO2 in the brain, acute coronal brain slices were prepared and imaged after bath application of CTZ. Bioluminescence was detected throughout the basal ganglia circuitry (Fig. 5d), suggesting widespread activation of iLMO2. Post mortem histology confirmed that expression of iLMO2 was found throughout the cell bodies and projections of neurons in the globus pallidus and striatum (Fig. 5b). These results demonstrate iLMO2′s ability to modulate multiple circuits underlying complex behavior in freely moving animals in a minimally-invasive manner.

## Discussion

We report here a novel optogenetic probe that is capable of suppressing neural activity in response to both external light and chemical substrate. In contrast to previously described excitatory luminopsins that were shown to modulate neuronal excitability *in vitro*^12^, we have demonstrated robust inhibition of action potential firing in various scenarios *in vitro* and *in vivo*. First, we rationally designed inhibitory luminopsins by characterizing the bioluminescence emission properties of various luciferase proteins and selecting the most suitable ones to couple with an inhibitory opsin, NpHR. We then demonstrated that bioluminescence from the *Renilla* luciferase variant, Nano-lantern, could be used to activate NpHR when both are coupled together as the iLMO2 fusion protein or co-expressed in the same cell (Fig. 1). Although co-expression of opsin and luciferase uniquely allows for the possibility of transcellular optogenetic modulation if expression is spatially targeted to different cells, we pursued the single fusion protein approach due to its more effective transgene delivery and greater coupling efficiency. iLMO2 was then utilized to suppress action potential firing and synchronous bursting activity *in vitro* (Figs 2 and 3). These effects were translated *in vivo*, where bimodal activation of iLMO2 was able to suppress single-unit firing rate and local field potential in the hippocampus of anesthetized rats (Fig. 4). Finally, iLMO2 was used for hardware-independent optogenetic inhibition of the basal ganglia, where its effects increased ipsilateral rotations for more than one hour in awake behaving rats (Fig. 5).

We have bypassed the challenges of delivering external light into the brain by making opsin-expressing cells their own light source with the use of bioluminescent proteins. The major advantage of utilizing luminopsins is that both the light source and opsin are genetically encoded and expressed as one molecule, which allows for modulating neural activity without the need for invasive hardware, as well as the ability to selectively target expression in a readily scalable manner. We demonstrated these advantages by modulating behavior in freely moving rats without the need for any chronically implanted devices or external light sources. Our results demonstrate that large structures in the brain can be targeted with luminopsins, which can be advantageous when multiple brain regions or diffuse circuits need to be manipulated. Indeed, expression of iLMO2 was found in both cell bodies and projections of the striatum and globus pallidus in animals used for the rotation tests, which suggests that inhibition of the direct pathway (striatal-nigral) and disinhibition of the indirect pathway (pallido-subthalamic) acted synergistically to modulate rotational behavior. Expression of iLMO2 was estimated to span a total volume greater than 20 mm^3^ in the brains of these animals, which is far greater than the predicted volume of light illumination achieved with a single optical fiber (the predicted spherical volume illuminated with at least 3.5 mW/mm^2^, an intensity shown to reliably activate NpHR^26^, is 1.15 mm^3^ using a 630-nm, 20 mW output, 200 μm core diameter, 0.39 NA fiber^27^).

Although we were able to influence a large volume of tissue in the brain by delivering iLMO2 with a relatively non-specific viral vector, one could also spatially restrict the expression of luminopsin to genetically defined cell populations with the use of cell-specific promoters or transgenic animals. In addition, luciferase-derived bioluminescence can also be spatially localized within the cell with the use of various targeting motifs^28^, which could potentially be useful for limiting optogenetic manipulation to subcellular components or preventing off-target activation of surrounding opsin-expressing cells. A genetically encoded luminopsin can therefore achieve high spatial resolution when expression is carefully controlled (*e.g.* with cell-specific promoters, localization motifs, or precise virus injections). Utilization of a genetically encoded light source can also yield distinct advantages over external light sources particularly when targeting complex structures that are problematic for efficient light transmission^8^ (*e.g.* cerebellum) since the light source is directly coupled to the opsin.

iLMO2 was able to generate enough hyperpolarizing photocurrent to completely suppress neural activity *in vitro*, but was only able to suppress single-unit firing rate by 60.3% on average *in vivo*. Our data suggests that this degree of inhibition is underestimated because single-unit firing rates actually increased after vehicle was injected. This increase in firing rate was likely not directly due to the injection itself, but rather because baseline activity gradually increased as animals were kept under constant isoflurane anesthesia. Indeed, recordings from animals on low levels of isoflurane (0.5–1.5%) showed a gradual increase in hippocampal activity over time (Supplementary Figure 2), which agrees with previous findings demonstrating the excitatory effects of low dose isoflurane on hippocampal activity^29^,^30^. Despite this excitatory effect of isoflurane, CTZ was still able to suppress single-unit activity just as well as 620-nm light delivered by optical fiber. The challenges of delivering external light into the brain were markedly illustrated by the fact that luciferase-derived light actually outperformed external light of the same peak wavelength (525-nm) given from the same location in the brain. The utilization of brighter and more red-shifted luciferases may help improve the performance of iLMO2 further, since better spectral overlap with NpHR and greater tissue penetration with longer wavelength light could improve both the coupling efficiency and volume of activation, respectively.

Luminopsin activity is contingent on the presence of either externally delivered light or luciferase substrate. Luminopsins therefore have a wide dynamic range of temporal control due to two modes of activation: temporally precise activation (on the order of milliseconds) with external light to more gradual and longer lasting activation (on the order of minutes) with luciferase substrate. Luminopsin activity triggered by either of these modes can also be temporally tuned based on the amount of light or substrate administered. The mode of activation can therefore be selected at the discretion of the user, where one mode can have substantial advantages or disadvantages over the other depending on the experimental context. For example, the gradual off-kinetics of the chemically activated approach can be leveraged to avoid the risk of rebound excitation that is exhibited by NpHR with conventional photostimulation^31^. Combining both modes of activation can create even more intricate duty cycles where external light can potentially add spatially and temporally precise periods of optogenetic manipulation over a network state primed by luciferase substrate. Since the modes of luminopsin activation can be both wide-acting and spatially precise, they could be appropriately utilized together for determining the relationship between global network activity (altered by luciferase substrate)and local activity of a subpopulation of neurons (altered by external light). These types of experiments would be more readily realized *in vivo* compared to other approaches utilizing multiple fibers or different combination of molecules^32^ since only one single molecule is needed.

The chemical-genetic approach to light production by luminopsins is reminiscent of DREADD (Designer Receptors Exclusively Activated by Designer Drugs) technology^33^,^34^, where a physiologic actuator (G protein in the case of DREADDs, opsin in the case of luminopsins) is specifically activated by an otherwise biologically inert molecule (CNO for DREADDs, CTZ for luminopsins). Although DREADDs have been shown to alter neural activity *in vitro* and *in vivo*, they face several unique challenges that are not necessarily shared with the luminopsin approach. First, DREADDs rely on secondary endogenous signaling proteins (*e.g.* G proteins), which are not necessarily present or active in every target population of neurons. In contrast, everything required to manipulate neural activity is encoded in a single molecule for the luminopsin approach. Second, these signaling proteins are involved in a variety of endogenous cell signaling pathways. The downstream effects of DREADD activation can therefore have very diverse and unintended effects other than altering membrane conductance compared to opsins. Third, only a single DREADD ligand (CNO) currently exists, which prohibits multiplexing different DREADDs for both excitation and inhibition in the same experimental paradigm. In contrast, a vast number of luciferases (each with their own spectral properties and substrate specificity) currently exist and can be coupled to compatible opsins. For example, CTZ-h specifically generates bioluminescence with *Renilla* luciferase but not *Gaussia* luciferase (which can only catalyze native CTZ^35^). Thus, iLMO2 can be potentially employed together with a *Gaussia* luciferase-based excitatory luminopsin (*i.e.* LMO1 and LMO2^12^) without the risk of crosstalk. Fourth, CNO undergoes back-metabolism to clozapine in humans which has multiple targets and effects in the brain, making it prohibitive for clinical translation^36^. Although CTZ has been widely used for *in vivo* imaging studies in rodents, no adverse effects of CTZ or its metabolite have been reported. Lastly, luminopsins may not necessarily suffer the same degree of limited temporal control seen with other chemical-genetic approaches. With the advent of activity-dependent luciferases (*e.g.* calcium dependent photoproteins like Aequorin^37^), luminopsin activity may be made responsive to neural activity or other biological cues in a closed-loop fashion and thus be activated only when it is needed. The luminopsin approach is thus unique in the fact that it combines the advantages of optogenetics (*i.e.* versatile and robust modulation of neural activity) with those of the DREADD approach (*i.e.* non-invasive chemical activation) into one single molecule capable of enabling responsive control of neural activity.

The luminopsin approach can be readily applied to existing and new optogenetic probes as they are continually developed. With the advent and discovery of new luciferases and opsins (each with their own different kinetics and spectral properties), much freedom and versatility exists in expansion of the luminopsin toolbox.

## Methods

### Plasmid construction

All constructs were initially cloned into the pcDNA3 backbone (Invitrogen) for ease of comparison. The luciferase constructs (TagRFP-Rluc8.6^38^ was a gift from Dr. Sanjiv Gambhir; Firefly luciferase^15^ was a gift from Dr. Phil Sharp, Addgene plasmid #11510; Nano-lantern^14^ was a gift from Dr. Takeharu Nagai, Addgene plasmid # 51970) were all PCR amplified and cloned into the pcDNA3 vector for direct comparison of expression and bioluminescence. The NpHR coding sequence was PCR amplified from the pAAV-eNpHR3.0-EYFP plasmid (gift from Dr. Karl Deisseroth) and inserted into the PCDNA3 vector. The EYFP coding sequence was digested out of this plasmid using EcoRI/NotI restriction sites to leave a backbone containing NpHR. iLMO1 was created by PCR amplifying out the TagRFP-Rluc8.6 cassette and inserting it in-frame downstream of NpHR. iLMO2 was created in a similar fashion, where the Nano-lantern cassette was PCR amplified (Forward primer: 5′ ATCGGAATTCGTGAGCAAGGGCGA, Reverse primer: 5′ ATCGCTCGAGTTACACCTCGTTCTCGTAGCAGAACTGCTCGTTCTTCAGCAC) and cloned downstream of the NpHR coding sequence. Both constructs also included an ER-export sequence^39^ at the 3′ end of the fusion protein. The iLMO1 and iLMO2 cassettes were subsequently cloned into the FUGW lentiviral backbone and the pAAV backbone for production of 2nd generation lentivirus and AAV2/9, respectively.

### Coelenterazine preparation

Coelenterazine-h was purchased from Promega and was solubilized in 20 mM β-cyclodextrin (Sigma) in PBS as described by Dr. Osamu Shimomura^40^. Frozen stock aliquots were kept at −20 °C and protected from light. For rotational experiments, CTZ was purchased from Nanolight and solubilized in their proprietary Inject-a-lume solvent immediately before use.

### Cell culture and transfection

HEK293 cells were grown in Dulbecco’s Modified Eagle Medium (DMEM) containing 10% fetal bovine serum (FBS) and 100 U/mL Penicillin/Streptomycin at 37 **°**C with 5% CO_2_, and were regularly passaged with 0.25% Trypsin/EDTA every 3–4 days. HEK293 cells were transfected with plasmids using Lipofectamine 2000 (Invitrogen) following manufacturer recommended protocols.

Cortical neuron cultures were derived from E18 rat embryos. Cortical tissue was digested with Papain (2 mg/mL) and mechanically dissociated by trituration with a fire-polished glass pipette. Dissociated cortical neuron cultures were seeded on poly-D-lysine coated 18 mm glass coverslips (NeuVitro) and were grown in Neurobasal media containing 1% FBS and 1X Glutamax (Invitrogen). The media was fully changed the day following plating with Neurobasal media containing B27 supplement and 1X Glutamax. Neuronal cultures were typically transduced with viral vectors 1 day *in vitro* at a multiplicity of infection (MOI) greater than 10 to ensure near 100% transduction efficiency. Half-volume media changes were subsequently given every 3–4 days.

### Viral vector production

iLMO2 lentivirus driven by the ubiquitin promoter (lenti Ub-iLMO2) was made in-house based on methods described by Dr. Trono Didier^41^. In brief, 293FT cells were grown in multiple 10 cm cell culture dishes to 90% confluency on the day of transfection. Each dish was then transfected with 7.5 μg Δ8.9 packaging vector, 3 μg VSVG envelope vector, and 10 μg of the iLMO2 transfer vector by calcium phosphate precipitation. Transfection efficiency was confirmed the following day by fluorescence microscopy and lentivirus was subsequently harvested for 2 days following transfection. The harvested lentivirus was purified through a 0.45 μM PES filter and concentrated by ultracentrifugation to an approximate titer of 10^8^ infectious particles/mL. AAV2/9 CAMKIIα-iLMO2 was produced by the Emory viral vector core at a titer of 10^12^ viral genomes/mL.

### Plate reader assay

For direct comparison of bioluminescence intensity, HEK293 cells seeded at the same density were transfected with 200 ng of luciferase construct (all in the PCDNA backbone) in triplicate wells of a 96-well white luminescence plate (Costar). Total luminescence was detected with a commercial plate reader (BMG Labtech) after adding CTZ or D-luciferin (Sigma) to each well (final concentration of 20 μM). The same gain and integration times were used for measuring all wells. For estimation of emission spectra, luminescence was similarly measured with the use of a filter wheel equipped with 420-nm, 485 nm, 520-nm, 544-nm, 584-nm, 590-nm, and 620-nm bandpass filters.

### Intracellular recordings

Intracellular recordings were taken from 12 day old dissociated cortical neurons cultured on glass coverslips. The glass coverslips were transferred to an 18 mm low profile recording chamber (Warner Instruments) and perfused using a Quick Exchange platform (Warner Instruments) with external solution containing 135 mM NaCl, 5 mM KCl, 1 mM MgCl_2_, 2 mM CaCl_2_*2H_2_O, 10 mM HEPES, 10 mM Glucose. Whole cell patch clamp recordings were obtained with glass pulled recording pipettes with 5–10 mΩ resistance containing internal solution consisting of 120 mM potassium gluconate, 2 mM MgCl_2_, 1 mM CaCl_2_*2H_2_O, 10 mM EGTA, 10 mM HEPES, 2 mM ATP. Recordings were sampled at 10 kHz with an EPC-9 amplifier (HEKA) and Patchmaster software for online analysis and later exported to Matlab for offline analysis. Membrane potential was clamped to −60 mV for voltage clamp recordings while current was clamped at 0 ± 20 pA for current clamp recordings in order to maintain a resting membrane potential of −60 mV. Action potentials were evoked by either giving threshold-level (rheobase levels evoking spikes ~50% of the time) or supra-threshold level (spikes evoked 100% of the time) current injections. Bioluminescence imaging was concurrently taken throughout the whole cell recordings with a 1.35 NA oil immersion objective, 0.35x demagnifying lens, and a scientific CMOS camera (QImaging). CTZ was bath applied through a 4 channel micro-manifold (AutoMate Scientific) to reach a final concentration of 40 μM in the recording chamber. Cells were illuminated through the objective with green light (532–554 nm TRITC filter cube at 35 mW/mm^2^) using an electronic shutter. Percent inhibition of spikes were calculated as the peak reduction of firing rate across the recordings taken in 10 s bins.

### *In vitro* multielectrode recordings

High density cortical neuron cultures grown on multielectrode arrays were prepared and recorded using the same equipment and methods described by Hales *et al.*^42^. Briefly, approximately 50,000 dissociated cortical neurons were seeded in each well of a 6-well multielectrode array (Multichannel Systems). Each well contained 9 recording electrodes, in which multiunit activity was detected and acquired by a custom-built NeuroRighter data acquisition system^43^. The cultures were covered with a Teflon lid to prevent evaporation and kept in a humidified incubator set at 35 **°**C and 5% CO_2_. The cultures were infected 1 day *in vitro* with Ub-iLMO2 lentivirus at >10 MOI to ensure near 100% transduction efficiency, and the media was subsequently changed (half-volume) every 3–4 days with Neurobasal media containing 2% FBS. Approximately 2 weeks later, the MEA was connected to a pre-amplifier located inside an incubator kept at 65% humidity, 35 **°**C, and 5% CO_2_. Spontaneous bursting activity in the cultures was confirmed and the MEA was allowed to equilibrate in the incubator overnight. On the day of the experiment, 5 μl of CTZ was added to the media of each culture to reach a final concentration of 12 μM. Recordings were taken up to 6 hours after addition of CTZ and analyzed offline using custom Matlab scripts. Synchrony was calculated using custom Matlab scripts developed by Quiroga *et al.*^44^. Percent change in firing rate was calculated by averaging the firing rate of each electrode in the culture across a 10 min window immediately after and 6 hours after the addition of CTZ.

### Animal experiments

All animals were purchased from Charles River Laboratories and were housed in the Emory animal vivarium with a 12 hr light/12 hr dark cycle. All procedures were conducted in accordance to approved guidelines from the Emory University Institutional Animal Care and Use Committee.

### Stereotaxic viral injections

For animals implanted with a cannula-electrode, two month old male Sprague-Dawley animals (200–250 g) were anesthetized with 1.5–4% inhaled isoflurane and a craniectomy was made 3.3 mm posterior and 3.2 mm lateral to bregma. 1.8 μl of AAV2/9-CAMKIIα-iLMO2 was stereotaxically injected at a depth of 3.1 mm ventral to pia targeting the dorsal hippocampus. Virus was injected through a glass-pulled pipette using a Nanoject injector (Drummond Scientific) at a rate of 275 nl/min. After viral injection was completed, the scalp was stapled closed and animals were allowed to recover for up to two weeks.

For animals used in behavioral rotation tests, iLMO2 was unilaterally expressed in the striatum and globus pallidus of rats by two stereotaxic injections (−0.4 mm AP, ±3.5 mm ML, −5.0 mm SI; +1.0 mm AP, ±2.8 mm ML, −5.2 mm SI) of AAV2/9-CAMKIIα-iLMO2 as described above.

### *In vivo* electrophysiology

Animals were implanted with a cannula-electrode two weeks after the viral injection. We have found that two weeks is sufficient for expression of optogenetic vectors delivered by AAV. A guide cannula (Plastics One) was glued to a 16-channel microwire array (Tucker Davis Technologies) so that a 0.39 NA, 200 μm core diameter bare fiber optic (Thorlabs) inside an injection cannula could be inserted into the guide cannula and positioned 1–2 mm away from the electrode tips. Four skull screws and one cerebellar reference screw were implanted into each animal. A craniotomy was then made over the dorsal hippocampus (array angled 50**°** from midline and centered 3.5 mm posterior and 2.9 mm lateral to bregma) and the cannula-electrode was manually driven into the brain while continuously recording to ensure ideal placement of electrodes. The correct stop depth was determined by identifying responsive single-units after repeated optical stimulation (10 s continuous pulses) with green (625-nm, 2.8 mW from fiber tip) and orange (520-nm, 1.3 mW from fiber tip) light from externally coupled LEDs (Plexon). Electrophysiologic recordings were sampled at 25 kHz using our custom built NeuroRighter data acquisition system^45^. Local field potentials were bandpass filtered (1–500 Hz) from the raw signal and analyzed offline using custom Matlab scripts and the Chronux toolbox^46^. Single-units were detected from the bandpass filtered (500–5 kHz) signal and sorted offline using superparamagnetic clustering (Wave Clus) scripts developed by Quiroga *et al.*^47^. The entire cannula-electrode was then sealed in place with dental acrylic (Lang Dental), the optical fiber was retracted from the guide cannula and replaced with a dummy cannula, and the animal was allowed to recover several days before further experimentation.

### Intracerebral injections

After the animals recovered from surgery, their electrophysiological responses to light, CTZ, or vehicle were investigated. The chronically implanted cannula-electrode allowed for multiple trials to be conducted for each animal, where each trial consisted of an intracerebral injection of CTZ and vehicle (in alternating order) on one particular day. The animals were first lightly anesthetized by 0.5–1.5% inhaled isoflurane. Responsive units were then identified by optically stimulating through the guide cannula with an optical fiber coupled to a 625-nm orange and 520-nm green LED as described above. Only responsive units were used for subsequent single-unit analysis. The optical fiber was then removed from the guide cannula and replaced with an injection cannula connected to a Hamilton syringe containing either 2 μl of CTZ (600 μM) or vehicle (20 mM β-cyclodextrin in PBS). A baseline recording of at least 10 minutes was recorded before CTZ or vehicle was injected (over a period of ~5 minutes) into the brain parenchyma. After injection was completed, the animal was recorded under constant levels of anesthesia for up to 1 hr. Single-unit activity was normalized to the median firing rate 10 minutes before injection. Percent inhibition of spikes to CTZ was calculated by averaging the percent reduction in firing rate ±1 min from the point of maximum inhibition. Percent inhibition of spikes to vehicle injection was calculated in the same fashion using the same time interval used in the analogous CTZ calculation.

### Rotation tests

One week after virus injection, animals were implanted with a jugular venous catheter to allow for convenient intravenous injections of CTZ or vehicle during rotational testing. Therefore, each animal could undergo multiple trials of both CTZ and vehicle injections. At the start of each trial day (beginning in the mornings between 10–12pm), animals were given amphetamine (2.5 mg/kg) by intraperitoneal injection and placed into a cylindrical chamber where an automated rotation counter (Columbus Instruments) was used to quantify both partial and full rotations. When the animals reached a steady baseline, they were injected with either 250 μl of CTZ (1 mg) or vehicle through a syringe connected to the catheter by polyethylene tubing. The number of total rotations per two minute intervals was then recorded for up to 1.5 hours following injection.

### *In vivo* bioluminescence imaging

Bioluminescence imaging was conducted after the conclusion of the amphetamine-induced rotation tests. A craniotomy was first performed over the injection sites to allow for direct visualization of the intact brain and overlaying dura. The animals were then deeply anesthetized with 100 mg/kg Ketamine/Xylazine and injected with 0.5 mg of CTZ through the jugular catheter. Bioluminescence images were taken using 10–20 s exposure times in a Fuji LAS-3000 dark box equipped with a cooled CCD camera. The animal was subsequently sacrificed after the imaging session by Euthasol injection and acute brain slices were collected and imaged in a similar fashion (after bath application of CTZ to reach final concentration of 30 μM) using 1–2 s exposure times.

### Histology

After all experiments were completed, animals were sacrificed by intraperitoneal injection of Euthasol (Virbac). Animals were then transcardially perfused with 4% paraformaldehyde (PF). The heads of animals implanted with the cannula-electrodes were decapitated and kept in PF at 4 °C overnight to allow for visualization of the electrode tracks. Otherwise, the brains were dissected out and allowed to fix in PF for 1 hr. After fixation, the brains were cryoprotected in 30% sucrose before sectioning on the microtome. 50 μM thickness sections were collected and mounted onto glass slides for imaging on an upright fluorescence microscope using NIS-elements acquisition software (Nikon).

## Additional Information

**How to cite this article**: Tung, J. K. *et al.* Inhibitory luminopsins: genetically-encoded bioluminescent opsins for versatile, scalable, and hardware-independent optogenetic inhibition. *Sci. Rep.* **5**, 14366; doi: 10.1038/srep14366 (2015).

## Supplementary Material

Supplementary Information
