# Supplementary material for: Inhibitory luminopsins: genetically-encoded bioluminescent opsins for versatile, scalable, and hardware-independent optogenetic inhibition
Source: Sci Rep. 2015 Sep 24;5:14366. doi: 10.1038/srep14366 (PMC4585826; doi:10.1038/srep14366)
Supplement: Supplementary Information [file srep14366-s1.pdf]

**Supplementary Information**

**Title:** Inhibitory luminopsins: genetically-encoded bioluminescent opsins for versatile, scalable, and hardware-independent optogenetic inhibition.

**Authors:** Jack K. Tung[1,2], Claire-Anne Gutekunst[2], *Robert E. Gross[1,2,3]

[1]Coulter Department of Biomedical Engineering. Georgia Institute of Technology and Emory University. Atlanta, GA.

[2]Department of Neurosurgery. Emory University. Atlanta, GA.

[3]Department of Neurology. Emory University. Atlanta, GA

*Corresponding author

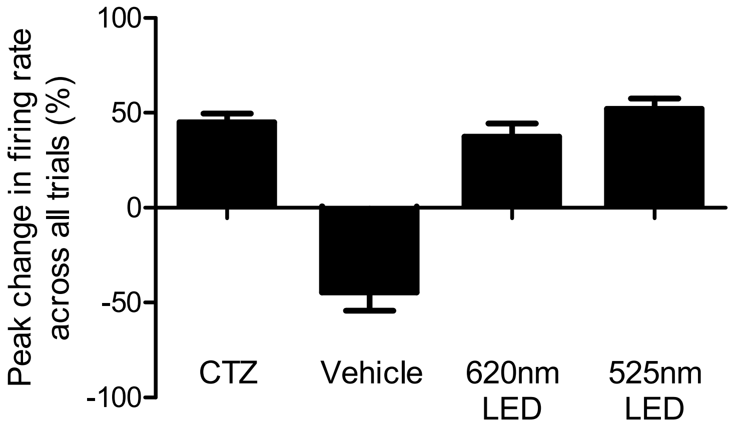


**Supplementary Figure 1:** Average peak change in single-unit firing rate across all trials of light stimulation, CTZ injections, and vehicle injections ($n$ = 5 trials each, 24 units across all trials) in the animal shown in Figure 4c-d.

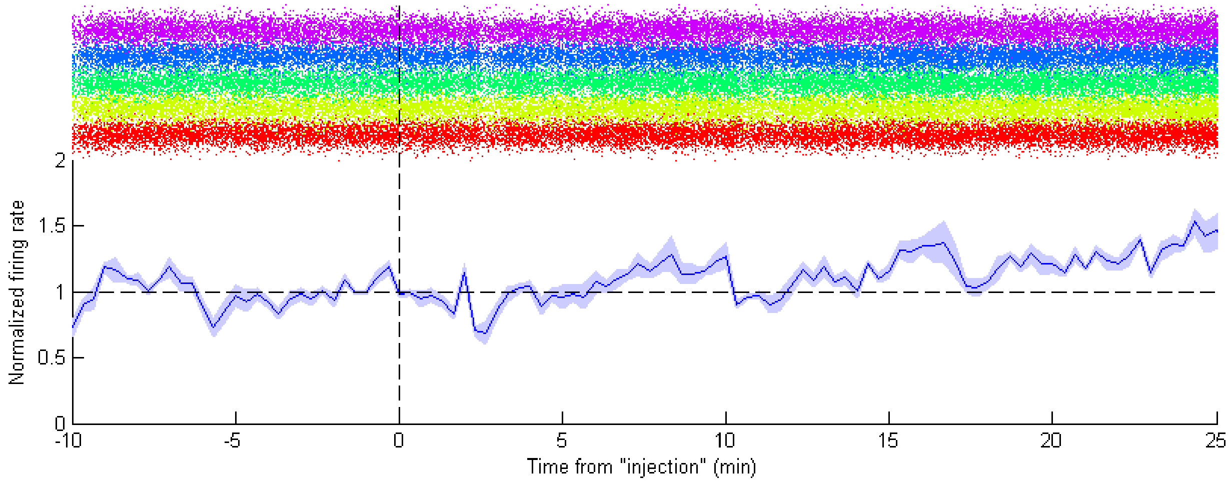


**Supplementary Figure 2: Hippocampal activity gradually increases under isoflurane anesthesia.** Normalized single-unit firing rate ($n$ = 5 units) over time for an animal on 1% isoflurane demonstrates a gradual increase in firing rate over time. Single-unit firing rate was similarly normalized to the median of a 10 minute baseline preceding an arbitrary "injection" time. SEM is shaded. Top: corresponding raster (each color corresponds to a different unit).
